# OX40/OX40L in systemic lupus erythematosus: Association with disease activity and lupus nephritis

**DOI:** 10.4103/0256-4947.75775

**Published:** 2011

**Authors:** Mohamed N. Farres, Dina S. Al-Zifzaf, Alaa A. Aly, Nermine M. Abd Raboh

**Affiliations:** a From the Department of Internal Medicine, Allergy and Clinical Immunology, Ain Shams University, Cairo, Egypt; b From the Department of Physical Medicine, Rheumatology and Rehabilitation, Ain Shams University, Cairo, Egypt; c From the Department of Medical Microbiology and Immunology, Ain Shams University, Cairo, Egypt; d From the Department of Pathology, Faculty of Medicine, Ain Shams University, Cairo, Egypt

## Abstract

**BACKGROUND AND OBJECTIVES:**

OX40-OX40L interaction is implicated in the pathogenesis of systemic lupus erythematosus (SLE). We evaluated the role of OX40/OX40L as markers of disease activity and nephritis in SLE patients.

**DESIGN AND SETTING:**

Case-control study conducted in 2009 on SLE patients attending the outpatient clinics of Ain Shams University Hospital, Egypt.

**PATIENTS AND METHODS:**

We assessed the percentage of CD4+ T-lymphocytes expressing OX40 by flowcytometry, and serum OX40 ligand (OX40L) levels in 40 patients with SLE (20 with lupus nephritis and 20 without) and in 20 healthy controls. Disease activity was assessed by the University of Toronto SLE disease activity index (SLEDAI).

**RESULTS:**

The percentage of CD4+ T-lymphocytes expressing OX40 was significantly higher in SLE patients than in controls, and in patients with lupus nephritis than in those without. OX40 expression correlated positively with both serum creatinine levels and SLEDAI. OX40 expression was the highest in patients with class V lupus nephritis and lowest in class II. Serum OX40L levels were significantly higher in SLE patients than in controls, and in patients with nephritis than in those without. Serum OX40L levels correlated with serum creatinine levels but not with SLEDAI. OX40 expression on CD4+ T-cells had a higher sensitivity and specificity in diagnosing lupus nephritis than both OX40L and anti–double-stranded DNA levels.

**CONCLUSION:**

OX40-OX40L interaction plays a role in the pathogenesis of SLE. The expression of OX40 on CD4+ T-lymphocytes and the serum level of OX40L may act as markers of lupus nephritis. Measurements of percentages of CD4+ T-lymphocytes expressing OX40 may serve as an indicator of disease activity in SLE.

OX40 (CD134) is a membrane-bound member of the tumor necrosis factor (TNF) receptor super-family, which can be found principally on activated CD4+ T-cells. The ligand for OX40 (OX40L) is expressed on activated antigen-presenting cells, including B-cells, macrophages, endothelial cells and dendritic cells.^1^

OX40-OX40L interaction has been implicated in the pathogenesis of autoimmunity. Engagement of OX40 on activated T-cells during antigen-specific T-cell stimulation can rescue effector T-cells from peripheral deletion. This results in a greater number of T-cells surviving the primary immune response and developing into memory T-cells, which may lead to the development of an autoimmune disease if they encounter their specific antigen.^2^

Furthermore, OX40L enhances B-cell proliferation and differentiation; thus its hyperexpression could augment the B-cell hyperactivity found in systemic lupus erythematosus (SLE). The resulting autoantibodies and immune complexes can mediate pathology in multiple systems in individuals with lupus. OX40L also negatively regulates the generation and function of IL-10–producing T-regulatory cells, which play a critical role in maintaining peripheral tolerance.^3^ Recent studies also found a region of OX40 that contains a risk haplotype for SLE, which is correlated with increased expression of cell-surface OX40.^4^

Lupus nephritis (LN) is one of the most severe complications of SLE and is characterized by the production of nephritogenic autoantibodies and immune complex formation.^5^ Observations in a mouse model of LN demonstrated the involvement of interactions between OX40 and OX40L in the development of glomerulonephritis.^6^ It was also reported that blockade of OX40 activation in animal models of autoimmune disease resulted in down-regulation of the inflammatory process. It was therefore proposed that the regulation of OX40-signaling may prove to be a beneficial and novel molecular target useful in the management of human inflammatory diseases.^1^ Studies on OX40 and OX40L in SLE in humans are rare. The aim of our study was to evaluate the role of OX40/OX40L as markers of disease activity and nephritis in SLE patients.

## PATIENTS AND METHODS

This case-control study was approved by the Review Board of the Rheumatology and Clinical Immunology departments, and was conducted on 40 patients with SLE attending the Rheumatology, Internal Medicine and Physical Medicine outpatient clinics of Ain Shams University Hospital, Egypt. All patients fulfilled the American College of Rheumatology (ACR) revised criteria for diagnosis of SLE.^7^ Patients with other rheumatologic diseases and nephritis due to other causes were excluded. Assessment of disease activity of SLE patients was done by the University of Toronto SLE disease activity index (SLEDAI).^8^ The study also included 20 age-grouped apparently healthy volunteers as controls, with no history of autoimmune diseases. They were selected from among relatives of patients admitted without autoimmune diseases. All subjects gave informed consent for participation in the study.

Serum levels of anti-nuclear antibodies (ANAs) were detected by indirect immunofluorescence (Euroimmun, Lübeck, Germany). Anti–double-stranded DNA antibodies (anti-dsDNA) were assessed by ELISA (Bio-Rad, Hercules, CA) and were considered positive if the antibody titer was ≥50 IU/mL. Complement C3 and C4 levels were measured by nephelometry (Dade-Behring, Marburg, Germany).

Renal functions were assessed by measuring serum levels of creatinine, blood urea, and creatinine clearance. Routine microscopic urinalysis and a 24-hour urine collection for protein assay were also performed. Nephritis was diagnosed if there was persistent proteinuria (more than 0.5 g/24 hours or more than 3+ by urine dipstick test) and/or cellular casts in urine (red blood cell, hemoglobin, granular, tubular or mixed). An ultrasound-guided renal biopsy was performed, after obtaining a written consent, for all SLE nephritis patients using a 16-gauge coaxial quick-core biopsy set. Light microscopy, electron microscopy and immunohistochemical staining were performed. Histopathological features of LN were classified by means of the International Society of Nephrology/Renal Pathology Society (ISN/RPS) classification of LN, 2003.^9^

OX40 and OX40L were assessed in all patients and controls. Four milliliters of venous blood were collected. Promptly separated serum was used for direct assay of soluble OX40 ligand by ELISA using Quantikine human OX40 Ligand Kit (RandD systems, Minneapolis, MN, USA), as recommended by the manufacturer. OX40L concentrations were expressed in pg/mL according to a standard curve. An additional 2 mL of blood was collected into tubes containing K-EDTA (1.2 mg/mL) for measurement of OX40 expression on peripheral blood CD4+ T-lymphocytes by flow-cytometry (Coulter EPICS XL). Samples were analyzed within 6 hours. Staining (triple-color surface staining) of 100 µL of each sample was done using 10 µL of each of fluorescein isothiocyanate (FITC), phycoerythrincyanin (Pecy5)-conjugated mouse monoclonal antihuman OX40 antibodies (Caltag Laboratories, Burlingame, CA), and phycoerythrin (PE)-conjugated mouse monoclonal antihuman CD4 (RandD Systems, Minneapolis, MN, USA). The tubes were then incubated in the dark at room temperature for 15 minutes. Erythrocytes were lysed using ammonium chloride lysing solution (Al-Gomhoreyya CA, Egypt). After two washes with phosphate-buffered saline (PBS), the cells were resuspended in PBS for flow-cytometric analysis. Negative isotype-matched controls were included with each sample to determine the nonspecific binding of the monoclonal antibodies. The results were expressed as percentage of the positive cells relative to the isotypic control.

Analysis of data was performed using the SPSS program, version 12. The t test was used to compare the means of two groups. The one-way ANOVA test was used to compare the means of more than two groups, followed by the Tukey test as a multiple comparison procedure to identify significant differences between means. Correlation (Pearson correlation coefficient r) assessing the strength of the relationship between two quantitative variables was performed. Correlations with anti-dsDNA were performed using the Spearman rank correlation coefficient, as anti-dsDNA levels were not normally distributed. Area under the curve (AUC) calculations of nonparametric receiver operating characteristic (ROC) curves were used to assess the sensitivity and specificity of OX40, OX40L and anti-dsDNA in diagnosing nephritis among patients with SLE. A P value of less than .05 was considered significant.

## RESULTS

This study was conducted on 40 SLE patients who fulfilled the ACR revised criteria for diagnosis of SLE. The SLE patients were divided into two groups according to the presence of nephritis. Group I included 20 patients with nephritis (18 females, 2 males; mean [SD] age, 27.2 [6.3] years, with a range of 17-42 years). Their mean (SD) disease duration was 5.6 (3.2) years (range, 1-11 years). Group II included 20 patients without nephritis (all females; mean [SD] age, 26.8 [9.5] years, with a range of 15-46 years). Their mean (SD) disease duration was 3.9 (2.9) years (range, 0.5-10 years). Twenty healthy volunteers were also included as controls (all females; mean [SD] age, 26.1 [7.8] years, with a range of 19-40 years). No significant differences in age or gender were detected between the three groups.

All 40 patients with lupus were positive for ANAs. Patients with LN had significantly higher SLEDAI, creatinine and anti-dsDNA levels than did patients without nephritis (**Table 1**). A higher percentage of CD4+ T-lymphocytes expressing OX40 was observed among SLE patients (21.01% [6.50%]) as compared to controls (7.21% [2.12%])—(t=9.21, *P*<.01). Expression of OX40 on CD4+ lymphocytes was significantly higher among SLE patients with nephritis than among those without nephritis. Furthermore, both patient groups showed significantly higher levels of OX40 expression as compared to controls (**Table 2**). Significantly higher serum levels of OX40L were detected among SLE patients (31.18[4.22 pg/mL]) as compared to controls (28.91[2.08 pg/mL])—(t=2.27, *P*<.05). Serum levels of OX40L were significantly higher among SLE patients with nephritis than among those without nephritis. Compared to the control group, only the SLE patients with nephritis showed significantly higher serum levels of OX40L (**Table 3**).

**Table 1:** SLEDAI, serum levels of creatinine, anti-dsDNA, C3, and C4 in the patient groups.

	Group I	Group II	*P*
SLEDAI	14.1 (3.52)	8.8 (3.58)	<.01
Creatinine (mg%)	1.78 (0.53)	0.78 (0.28)	<.01
Anti-dsDNA (IU/mL)	645, 404.75-918.75	290, 76.25-648.75	<.05
C3 (mg%)	59.8 (21.54)	72.3 (20.39)	>.05
C4 (mg%)	19.35 (7.06)	22.1 (8.87)	>.05

Values are mean (SD) except for anti-dsDNA, where values are median, interquartile range.

**Table 2:** Mean percentages of CD4+ T-cells expressing OX40 in the study groups.

	% of CD4+ T-cells expressing OX40 (mean [SD])	ANOVA	
		f	*P*
Group	25.30 (5.01)		
Group II	16.71 (4.77)	93.80	<.01
Controls	7.21 (2.12)		

Tukey test: *P*< .01 for Group I vs Group II, Group I vs controls, Group II vs controls.

**Table 3:** Serum-soluble OX40L levels in the study groups.

	Serum-soluble OX40L level (pg/mL) (mean [SD])	ANOVA	
		f	*P*
Group I	32.78 (4.69)		
Group II	29.59 (3.05)	7.19	<.01
Controls	28.91 (2.08)		

Tukey test: *P*<.05 for Group I vs Group II, *P*< .01 Group I vs controls, *P*> .05 Group II vs controls.

**Figure 1:**
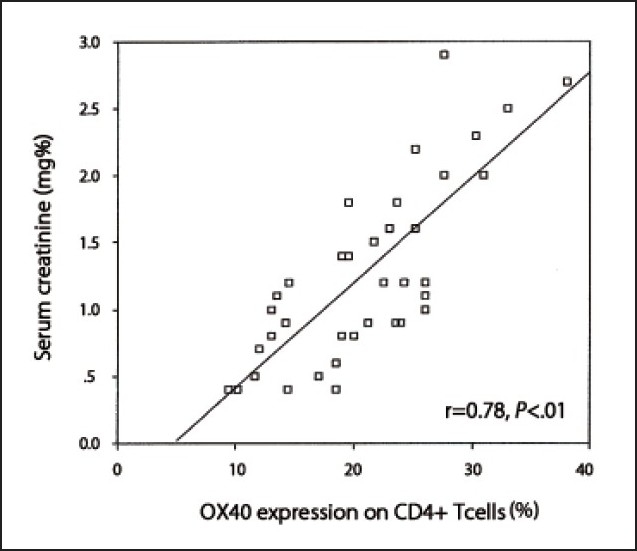
Correlation between OX40 expression on CD4+ T-cells and serum creatinine levels.

**Figure 2:**
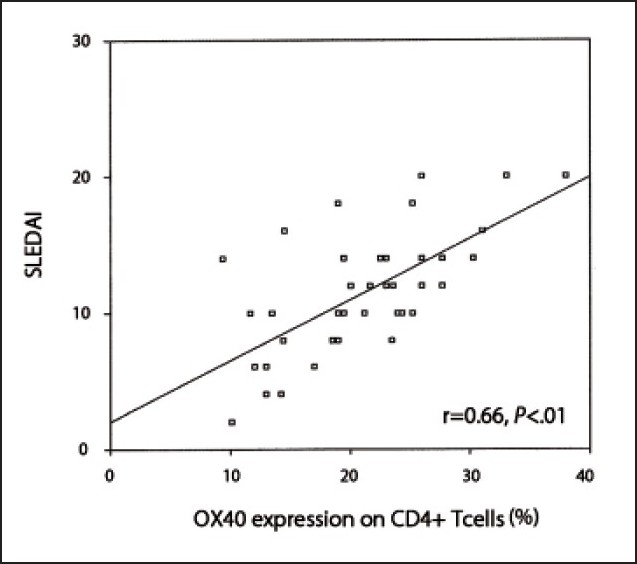
Correlation between OX40 expression on CD4+ T-cells and disease activity.

**Figure 3:**
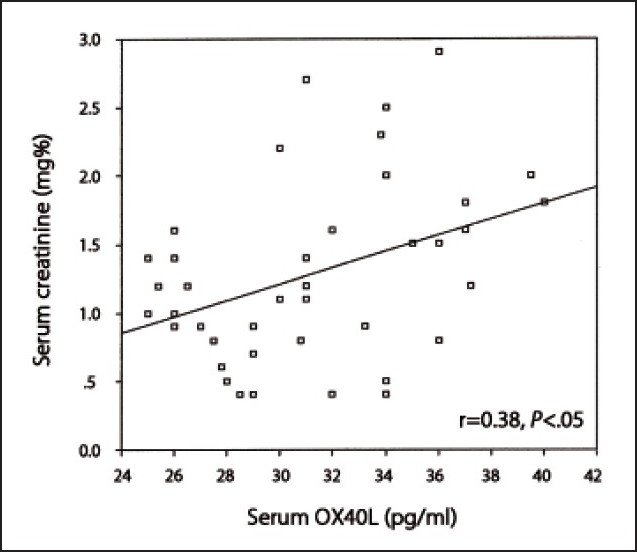
Correlation between serum levels of OX40L and creatinine.

Among SLE patients with nephritis, OX40 expression on T-lymphocytes was the highest among class V and lowest among class II. However, a less obvious trend was observed for serum levels of OX40L (**Table 4**). Among SLE patients, a significant positive correlation was observed between the percentage of OX40 expression on CD4+ T-cells and serum creatinine levels (r=0.78, *P*<.01) (**Figure 1**), and between OX40 expression and disease activity measured by SLEDAI (r=0.66, *P*<.01) (**Figure 2**). On the other hand, the serum levels of OX40L correlated significantly with serum creatinine levels (r= 0.38, *P*<.05) (**Figure 3**) but not with the disease activity as measured by the SLEDAI (r= 0.31, *P*>.05). Percentages of OX40 expression on CD4+ T-cells showed no significant correlation with anti-dsDNA, C3, or C4 levels (r=0.28, r=–0.19 and r=–0.24, respectively, *P*>.05). On the other hand, serum levels of OX40L correlated significantly with anti-dsDNA levels (r=0.36, *P*<.05), but not with C3 or C4 levels (r=–0.23 and r=–0.12, respectively, *P*>.05).

**Table 4:** OX40 expression on T-cells and serum levels of OX40L among the various classes of lupus nephritis in the study.

	Class of lupus nephritis			
	Class II (n=7)	Class III (n=6)	Class IV (n=5)	Class V (n=2)
% expression of OX40 on T-cells	22.07 (2.34)	23.70 (3.27)	28.06 (4.19)	34.50 (4.95)
Serum OX40L level (pg/mL)	32.14 (5.70)	31.20 (5.24)	34.56 (0.93)	35.25 (6.01)

Values are mean (SD).

**Figure 4:**
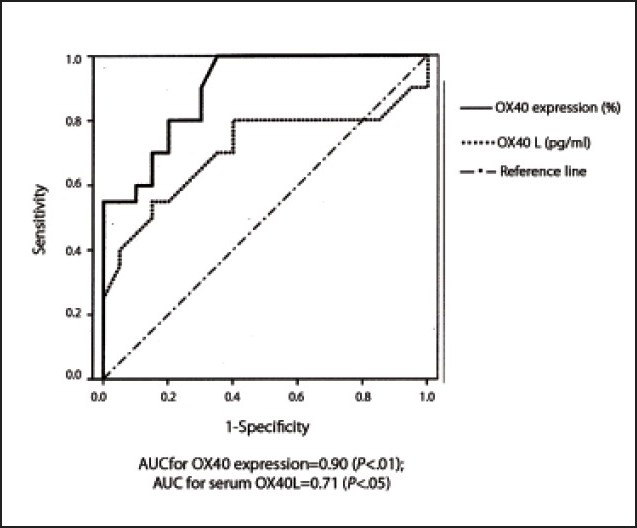
ROC curves for OX40 expression and serum OX40L, and the presence of nephritis in SLE patients.

To quantify the diagnostic utility of OX40, OX40L and anti-dsDNA as markers of nephritis in SLE patients, we constructed an ROC curve (**Figure 4**), using all markers in patients with LN proven by biopsy (the current gold standard) and patients without nephritis. At a cut-off value of 19.25%, the sensitivity of OX40 expression on CD4+ T-cells for the diagnosis of LN was 90%, and the specificity was 70%. The AUC was 0.90, with a 95% confidence interval of 0.81 to 0.99 (*P*<.01). On the other hand, we observed that serum OX40L levels at a cut-off value of 29.5 pg/mL had a sensitivity of 80% and a specificity of 60% for diagnosing LN. The AUC was 0.71, with a 95% confidence interval of 0.54 to 0.88 (*P*<.05). Serum anti-dsDNA levels had a sensitivity of 70% and a specificity of 65% for diagnosing LN at a cut-off value of 480 IU/mL. The AUC was 0.70, with a 95% confidence interval of 0.53 to 0.86 (*P*<.05).

## DISCUSSION

SLE is an autoimmune disease characterized by autoantibody formation, immune complex deposition and end organ damage.^10^ T-cells promote SLE in a variety of ways, including rendering help to B-cells for autoantibody production, as well as facilitating tissue damage in the end organs.^11^ Co-stimulatory molecules have a central role in driving T-cell activation in SLE. Abnormally increased expression of OX40 and/ or OX40L is a major reason for the activation of T-cells.^12^,^13^

In this study, we attempted to measure the percentage of CD4+ T-lymphocytes expressing OX40, to determine their role in the pathogenesis of SLE. Our results showed a highly significant increase in OX40 expression in SLE patients as compared to controls. We also found a highly significant positive correlation between disease activity assessed by the SLEDAI and the percentage of CD4+ T-lymphocytes expressing OX40. A previous study found an upgrade and clustering of OX40 on CD4+ T-cells in SLE, indicating its activation.^14^ Another study on experimental rabbits reported an improvement in SLE and a decline in the SLEDAI score upon administration of monoclonal antibodies against OX40.^15^ On the other hand, an earlier smaller study had reported that the difference between SLE patients and control subjects did not reach statistical significance.^16^ However, this could be attributed to the small number (8) of patients used in that study.

Patients with nephritis had a significantly higher percentage of CD4+ T-lymphocytes expressing OX40 than those without nephritis. Furthermore, OX40 expression correlated positively with serum creatinine levels and was the lowest in class II nephritis and highest in class V. This evidence suggests a role for OX40 in the pathogenesis of LN. Patschan et al^14^ demonstrated an up-regulation of OX40 on T-cells of SLE patients, with more clustering in those with active nephritis, although their results did not show any correlation between serum creatinine and OX40 expression, and they found similar percentages of expression among the different classes of nephritis. A recent study also showed that anti-OX40 therapy controlled the extent of T-cell–mediated damage in LN.^17^ CD4+ T-cells expressing OX40 could mediate LN either by aiding B-cells to produce antibodies, including anti-dsDNA antibodies, that may contribute to the kidney lesions^18^ ; or by directly infiltrating glomerular endothelial cells after ligation with OX40L, thereby causing direct damage.^19^

Serum-soluble OX40L was significantly higher in SLE patients than controls, although it did not correlate with disease activity. Furthermore, only patients with LN showed a significant increase in serum OX40L levels. Among patients with SLE, serum OX40L levels correlated positively with serum creatinine levels but did not vary greatly between the different classes of LN. A previous study reported an improvement in SLE after treatment with monoclonal antibodies against OX40L, with a decrease in the levels of IFN-gamma, IL-6 and anti-dsDNA antibody, which supports a role for OX40L in SLE pathogenesis.^13^ OX40L has been reported to be abundantly present in glomeruli in renal biopsies of almost all cases of proliferative LN, which supports a role for OX40L in LN.^16^ On the other hand, Patschan et al^14^ did not find any significant difference in serum-soluble OX40L concentration between SLE patients and healthy controls. This could be partially explained by the smaller number (27) of patients studied and the smaller proportion (8/27) of patients with LN.

The need for a reliable marker for LN is highly important. While renal biopsy is the gold standard for diagnosis and assessment of nephritis in patients with lupus, it is an invasive procedure that cannot be performed serially for monitoring purposes. Anti-DNA antibodies and complement levels often correlate with the presence of nephritis, but they are not specific and do not have a high discriminatory power. Their utility in reflecting disease activity and LN remains controversial.^20^ Our results showed that OX40 expression had higher sensitivity and specificity for diagnosing LN than both OX40L and anti-dsDNA, indicating its promising role as a marker of nephritis. This issue should be investigated in a study with larger cohort of patients, with special emphasis on the role of these markers in predicting the occurrence and severity of nephritis in SLE patients.

In conclusion, both OX40 expression on CD4+ T-cells and serum levels of OX40L may serve as markers of LN. Furthermore, measurements of the percentage of CD4+ T-lymphocytes expressing OX40 may serve as an indicator of SLE disease activity. We recommend further studies for immunohistochemical detection of OX40 and OX40L in renal biopsies in larger numbers of patients with LN, for further clarification of the pathology of LN. Therapeutic approaches with drugs that target these molecules might be useful in treatment of SLE and LN.
